# Supplementary figures and images for: A novel membrane complex is required for docking and regulated exocytosis of lysosome-related organelles in Tetrahymena thermophila
Source: PLoS Genet. 2022 May 19;18(5):e1010194. doi: 10.1371/journal.pgen.1010194 (PMC9159632; doi:10.1371/journal.pgen.1010194)

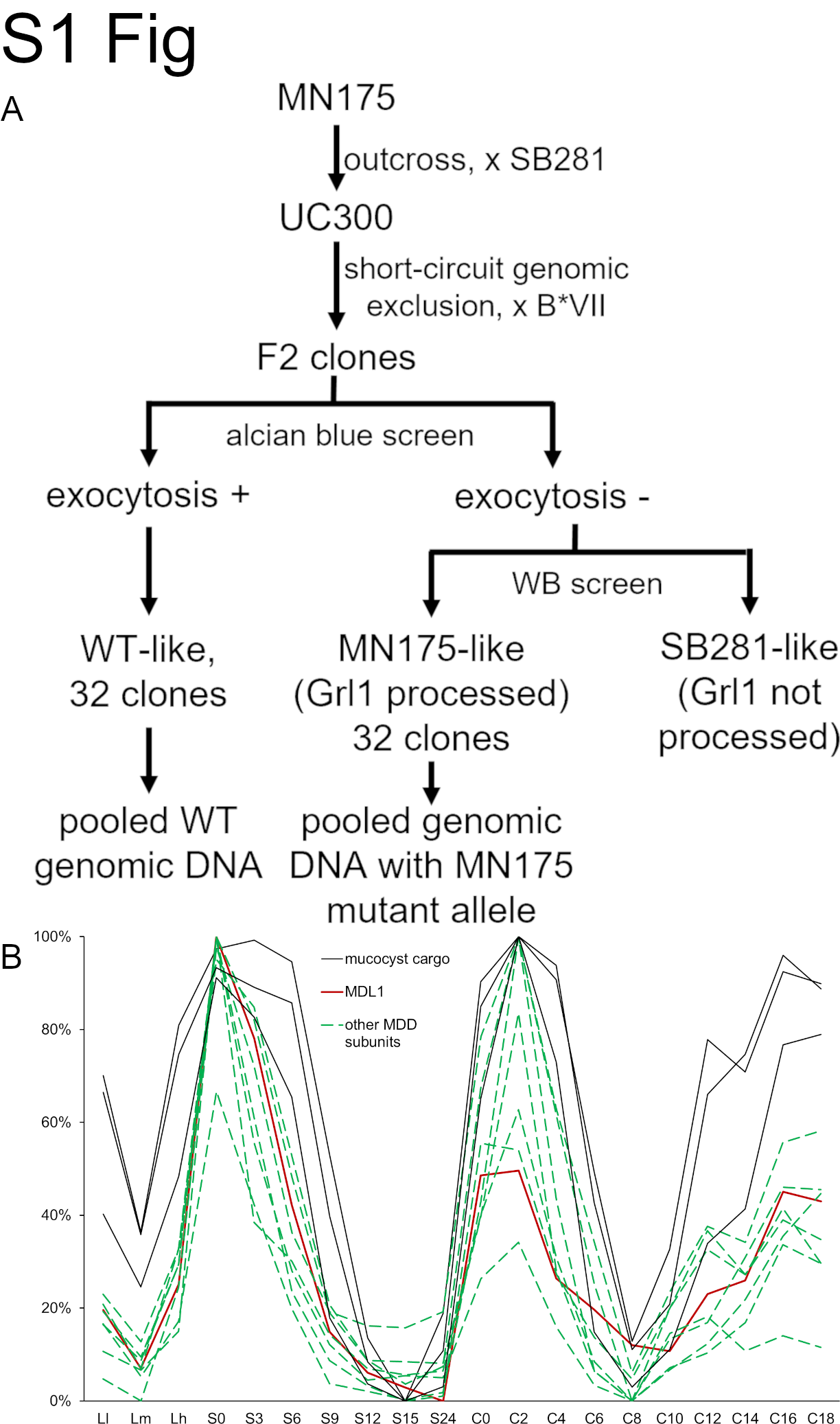

Supplement: S1 Fig — (A) Generating pooled genomic DNA of WT and MN175 clones for ACCA. MN175 mutant and UC300 strain used in this study were previously described. F2 clones of UC300 were generated and tested for exocytosis (see Materials and methods). Clones with defective exocytosis were further screened for processed Grl1p similar to Fig 1E. WT-like and MN175-like clones (32 each) were pooled to generated genomic DNA used in whole genome sequencing followed by ACCA. (B) Comparison of transcriptional profiles of MDL1 with genes encoding established mucocyst cargo proteins and proposed MDD subunits. Transcript levels are measured in growing cultures at three densities, followed by successive time points following starvation, and then during successive time points during conjugation, from tfgd.ihb.ac.cn. Mucocysts cargo protein genes include GRL1 (TTHERM_00527180), GRT1 (TTHERM_00221120) and IGR1 (TTHERM_00558350). Putative MDD subunits are listed in Fig 6B. Missing from the transcriptional analysis are TTHERM_000486279 and TTHERM_000193469, for which transcriptional profiles are not publicly available. (TIF) [file pgen.1010194.s001.tif]

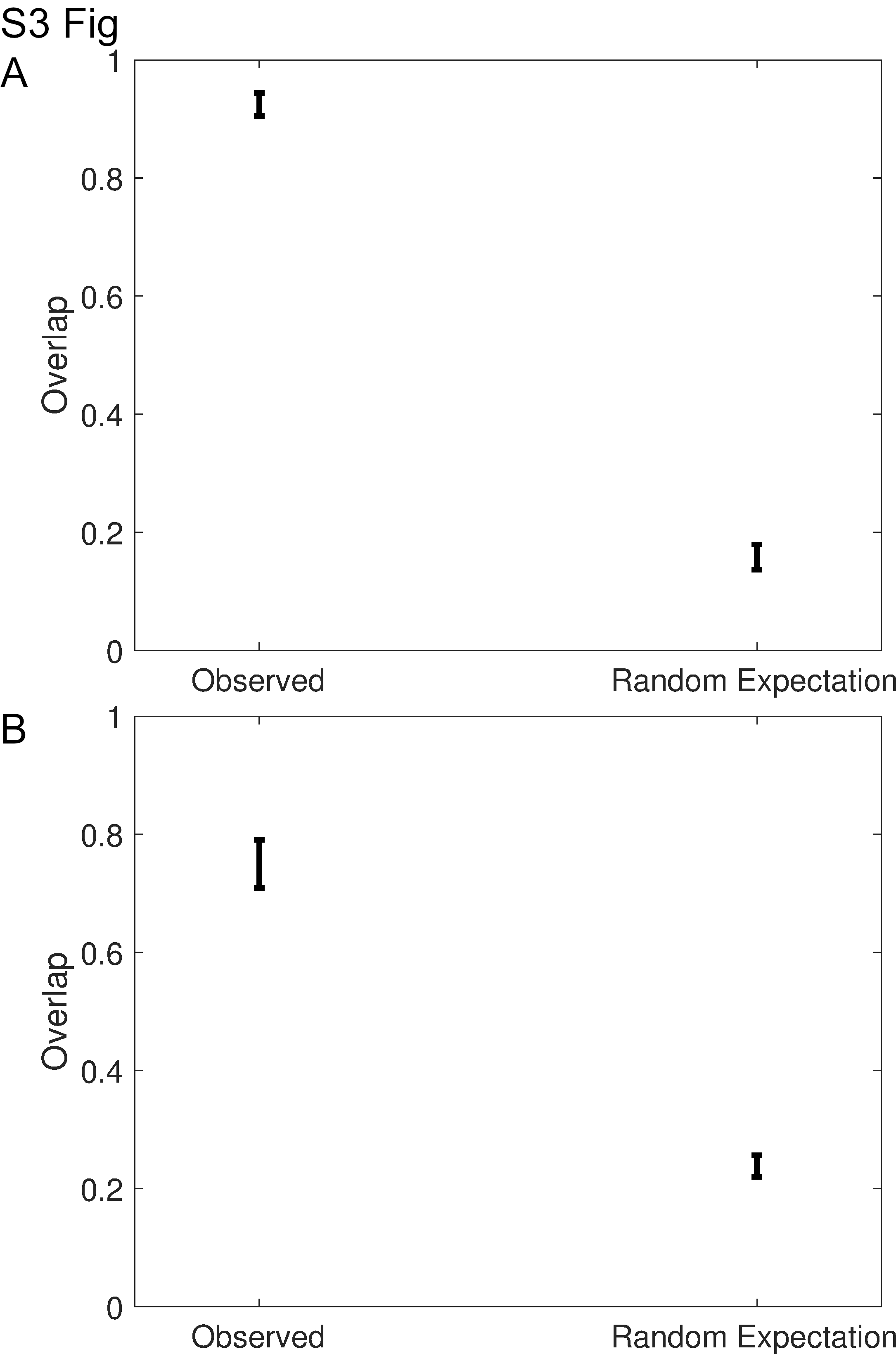

Supplement: S3 Fig — (A) The fraction of GFP-Mdl1p that overlaps with Grl3p in WT cells, at a sensitivity of adaptive thresholding appropriate to local maxima, compared to the overlap between randomized centroids within cell boundaries with Grl3p. Error bars indicate standard error of the mean (n = 6). (B) Same as A, but in MN173 cells. (TIF) [file pgen.1010194.s003.tif]

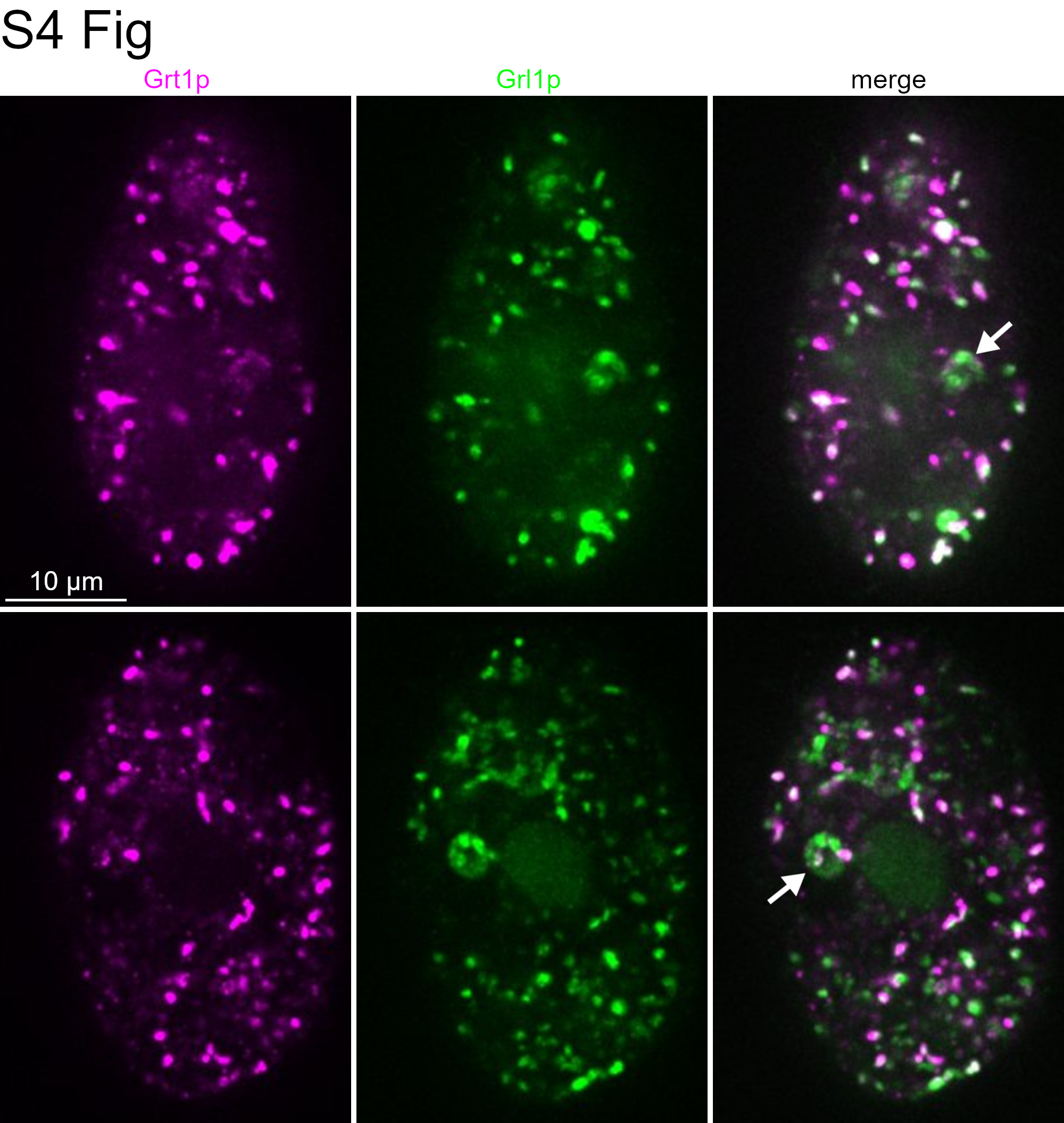

Supplement: S4 Fig — Undocked mucocysts appear to clump in Δmdl1. Starved Δmdl1 cells were immuno-stained with mouse Abs against Grl1p and Grt1p that were directly dye-coupled, as in Fig 4C. Shown are the individual channels and the merge. Clustering of mucocyst-related puncta (arrows) suggests they are incorporated in degradative bodies. Scale bar is indicated. (TIF) [file pgen.1010194.s004.tif]

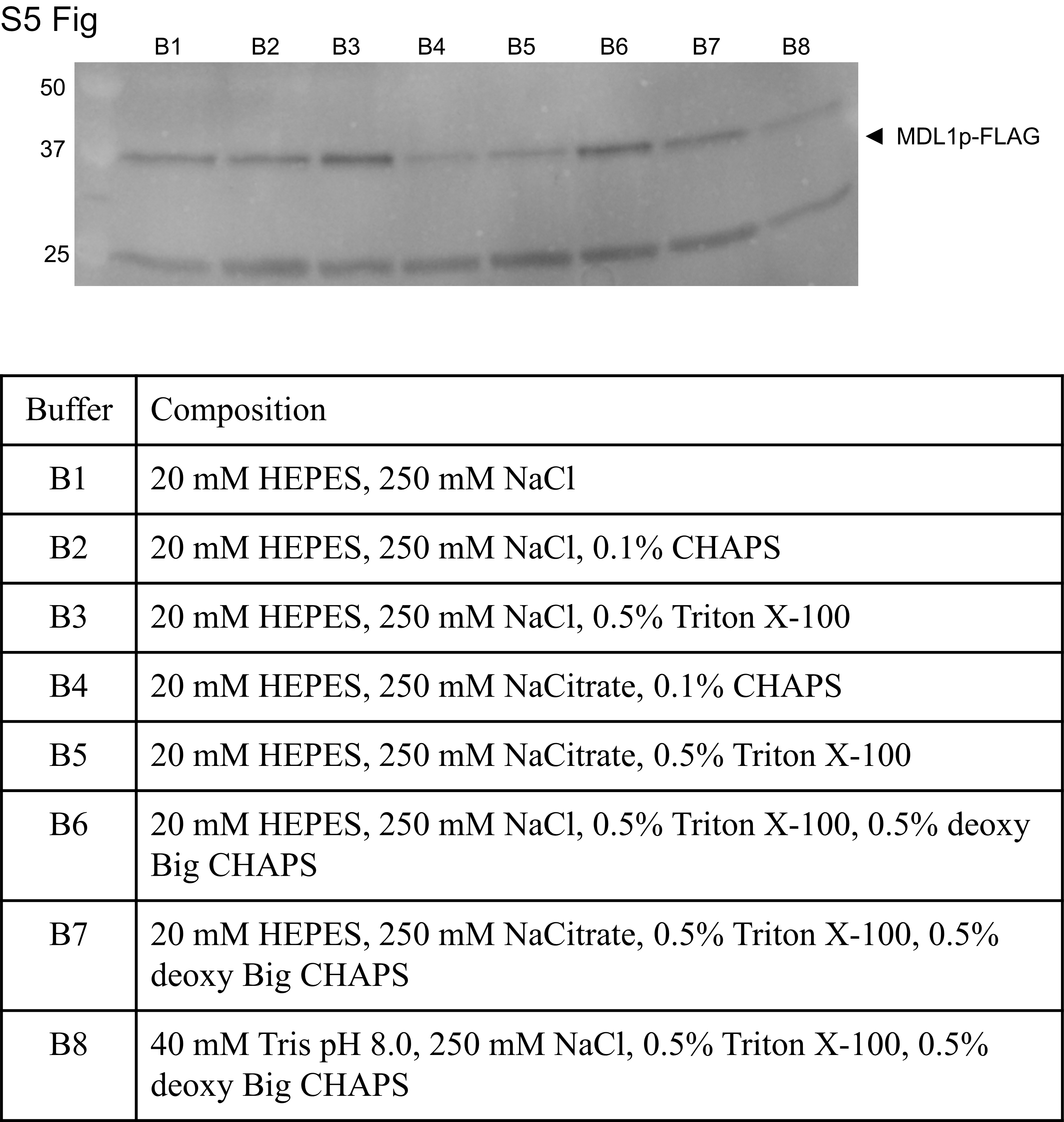

Supplement: S5 Fig — Testing of solubilization conditions for Mdl1p-FLAG from cryopowders. The solubilization assay was performed as described in Materials and Methods. Loaded in each lane is the soluble fraction of Mdl1p-FLAG, revealed by Western blotting with an anti-FLAG antibody, for the buffer composition shown in the table beneath. (TIF) [file pgen.1010194.s005.tif]

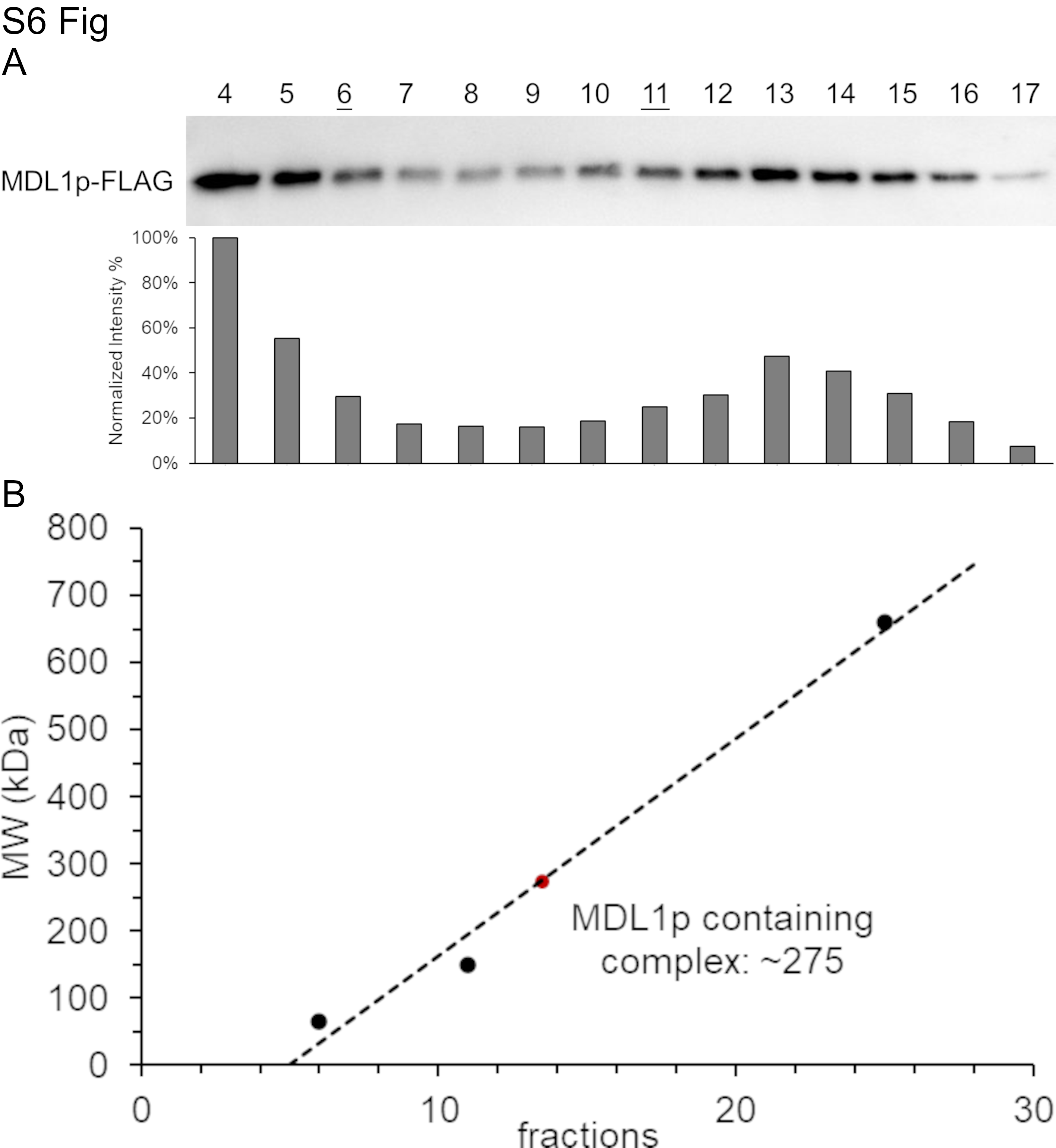

Supplement: S6 Fig — (A) Sedimentation analysis of Mdl1p. Mdl1p-FLAG, eluted with FLAG peptide from pulldowns, was sedimented on 10–40% glycerol gradients. Fractionated gradients were analyzed by SDS-PAGE followed by Western blotting with anti-FLAG antibody. Shown are fractions 4 to 17, in order of increasing density (of a total of 50 fractions). Fractions 4–5 and 13–14 contained the peak concentrations of Mdl1p-FLAG. Shown beneath are the relative intensities of the Mdl1p signals across the gradient fractions, normalized to the maximum after background subtraction. The peak fractions for size standards run on parallel gradients are indicated with underlining: BSA (66 kDa, fraction 6); yeast alcohol dehydrogenase (150 kDa, fraction 11); thyroglobulin (660 kDa, fractions 24–26). (B) Plotting the molecular weights of the standards as a function of their corresponding peak fractions suggests fraction 13–14 correspond to a molecular weight of 260–290 kDa. (TIF) [file pgen.1010194.s006.tif]
