# Supplementary material for: A novel membrane complex is required for docking and regulated exocytosis of lysosome-related organelles in Tetrahymena thermophila
Source: PLoS Genet. 2022 May 19;18(5):e1010194. doi: 10.1371/journal.pgen.1010194 (PMC9159632; doi:10.1371/journal.pgen.1010194)

S2 Fig

>TTHERM_00658810_MDL1

CTFSEIYAGAYQVARIQG

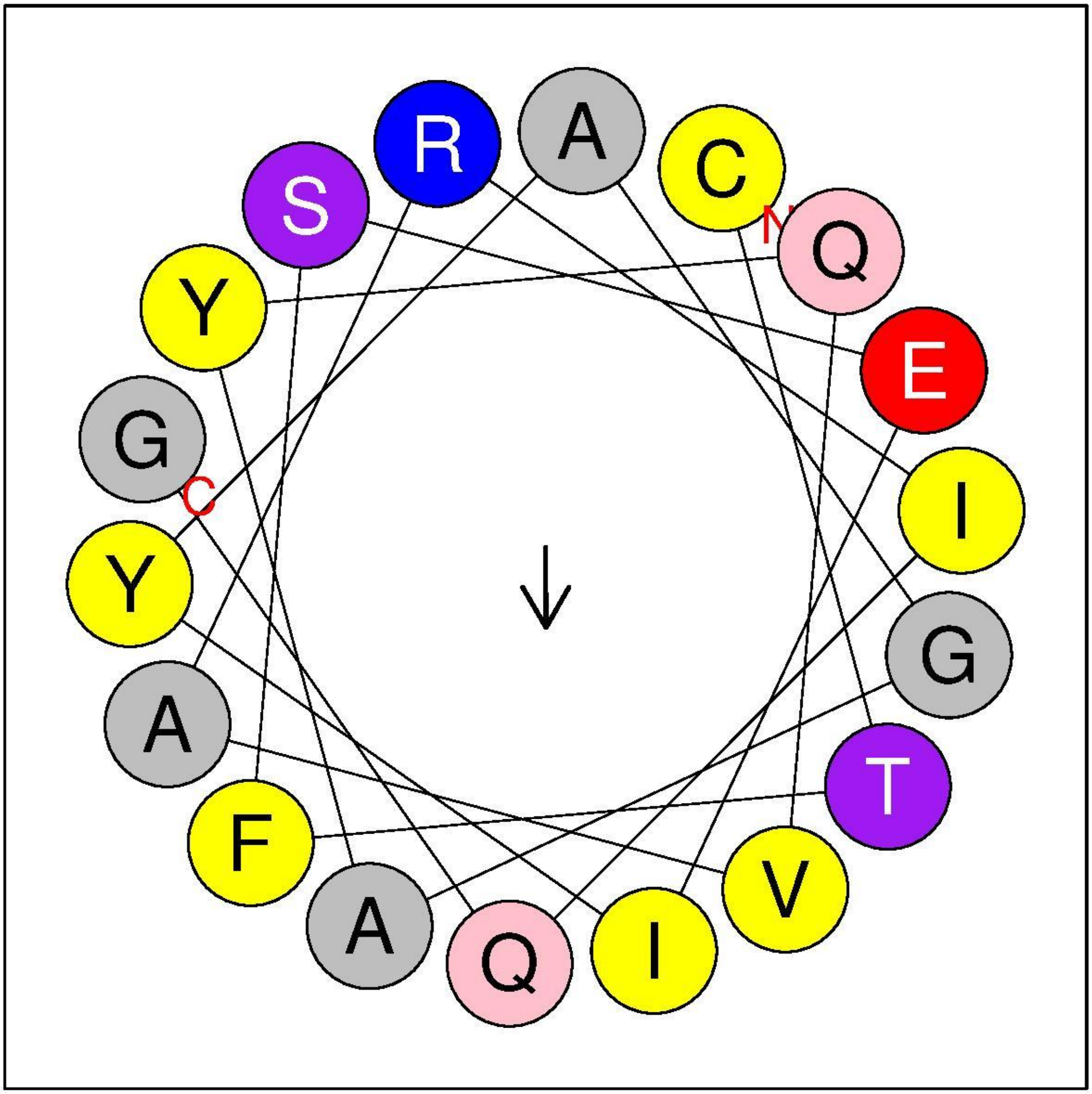

>TTHERM_000193469

LCTNLELYSGAYTIARFM

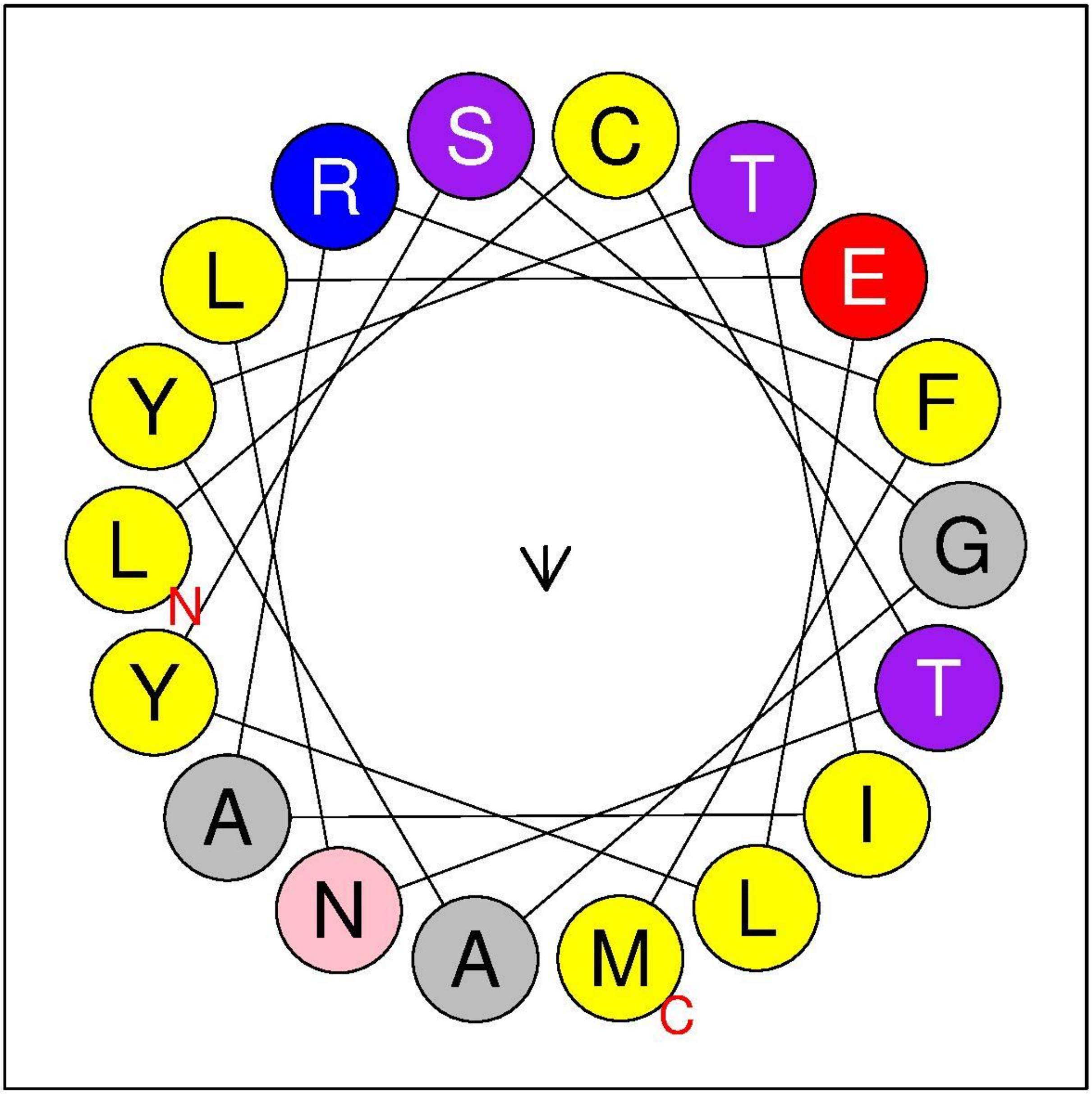

>TTHERM_00047330

CNSFEILSQGFQYARSMG

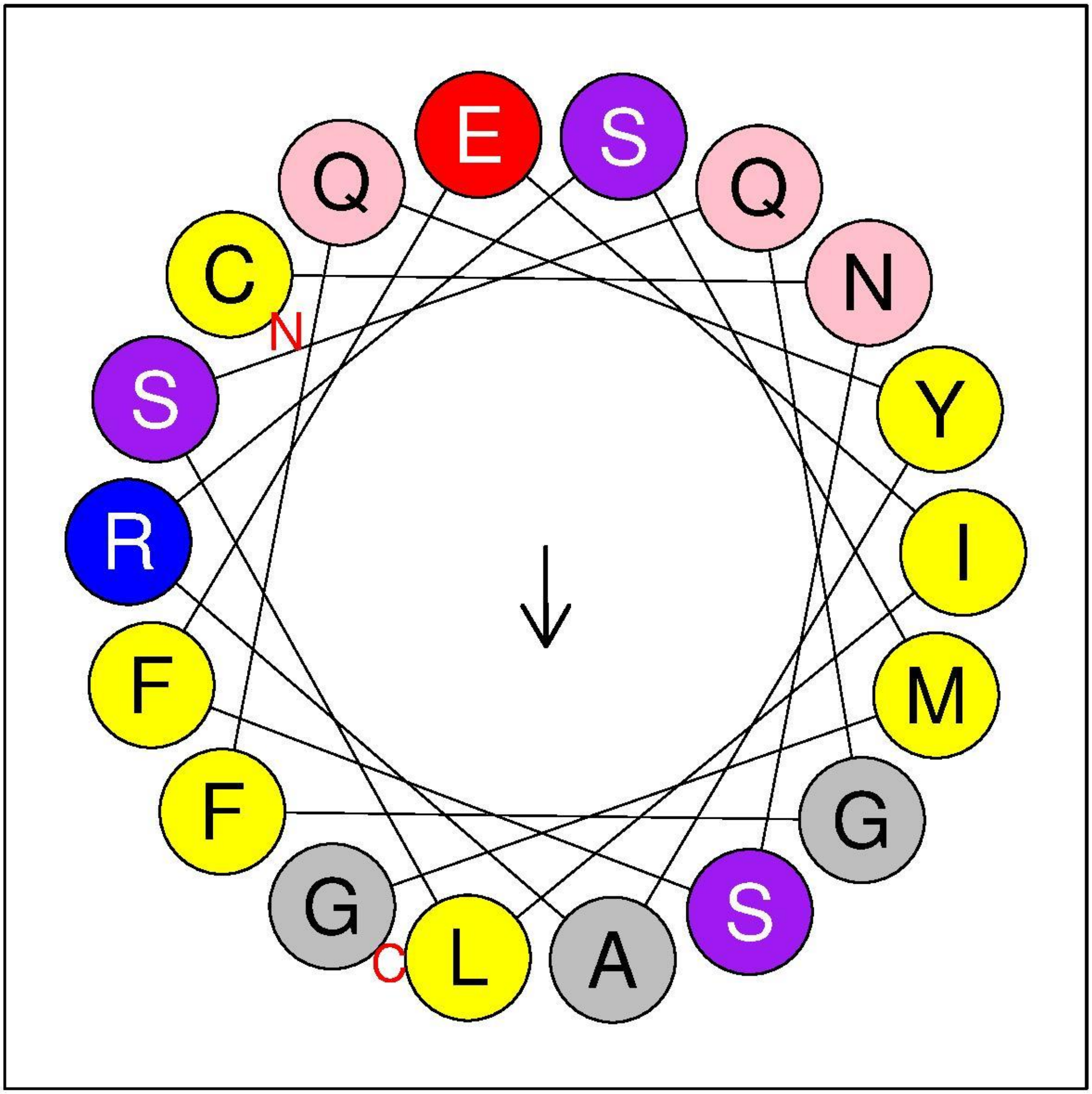

>TTHERM_000486279

ISFVVKHKLQSFEIELGL

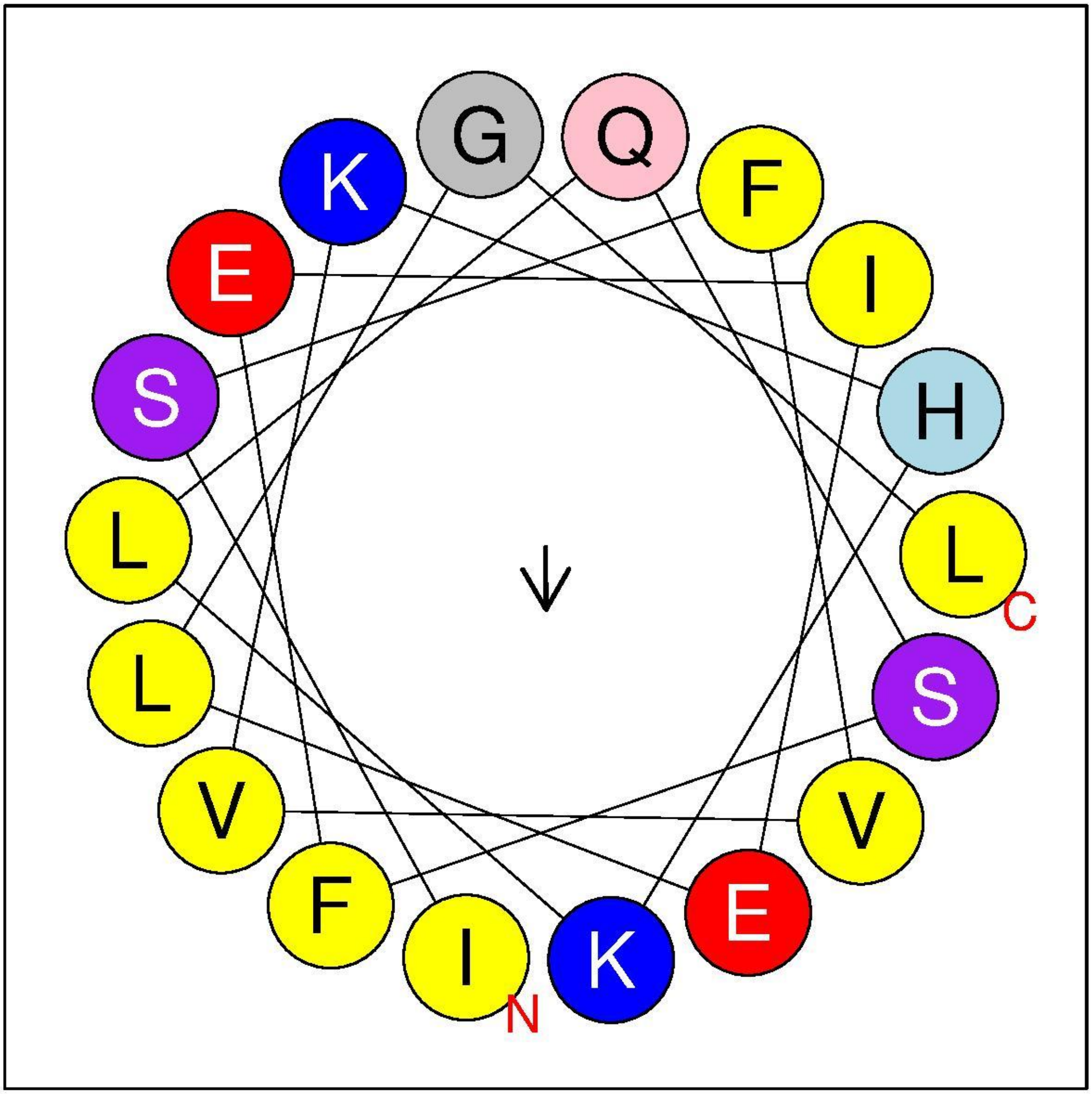

>TTHERM_00141040

YEGSFTHPPCTEGVTWII

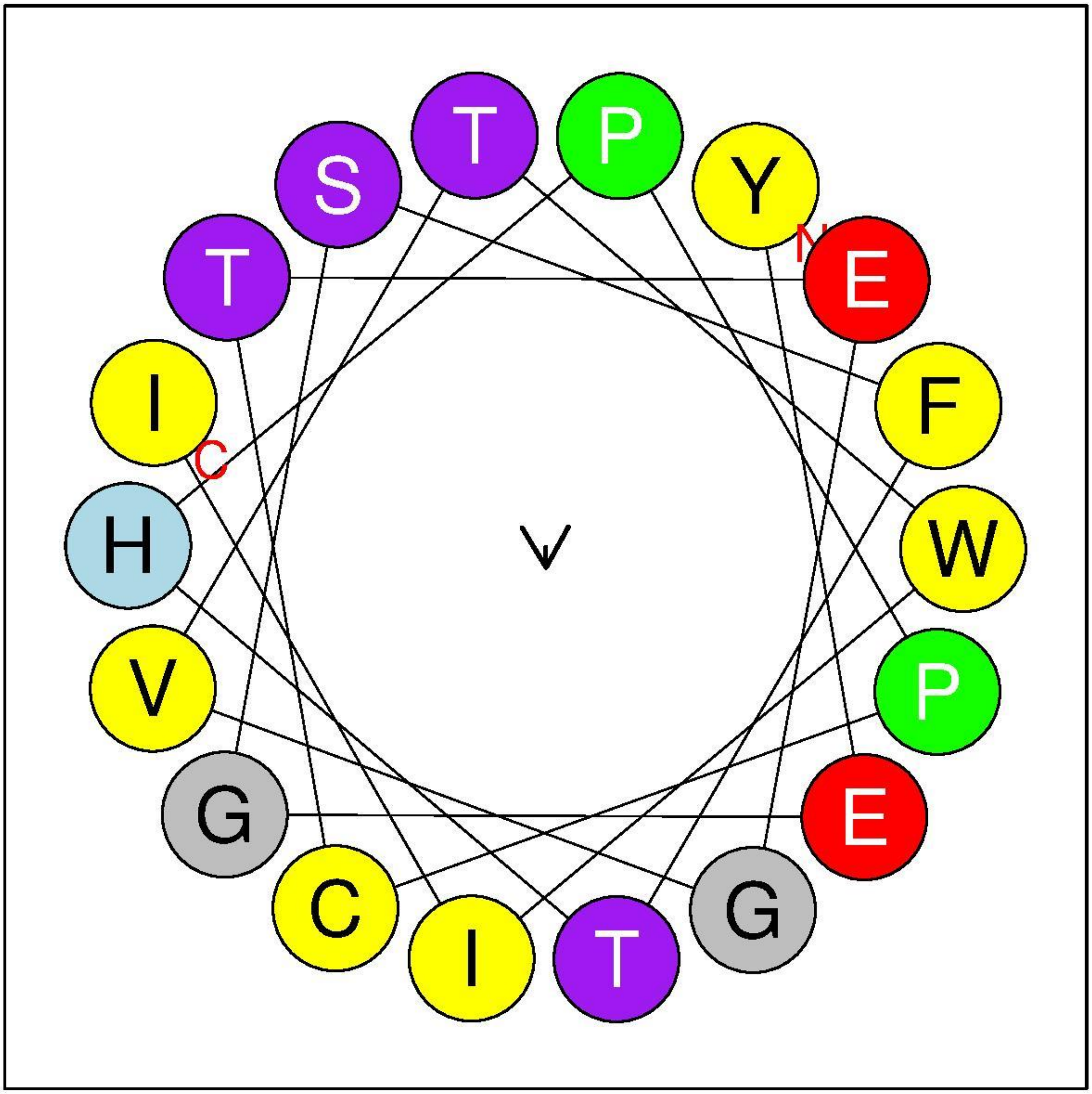

>TTHERM_00227750

ETEDPAEASFGLSNIQIY

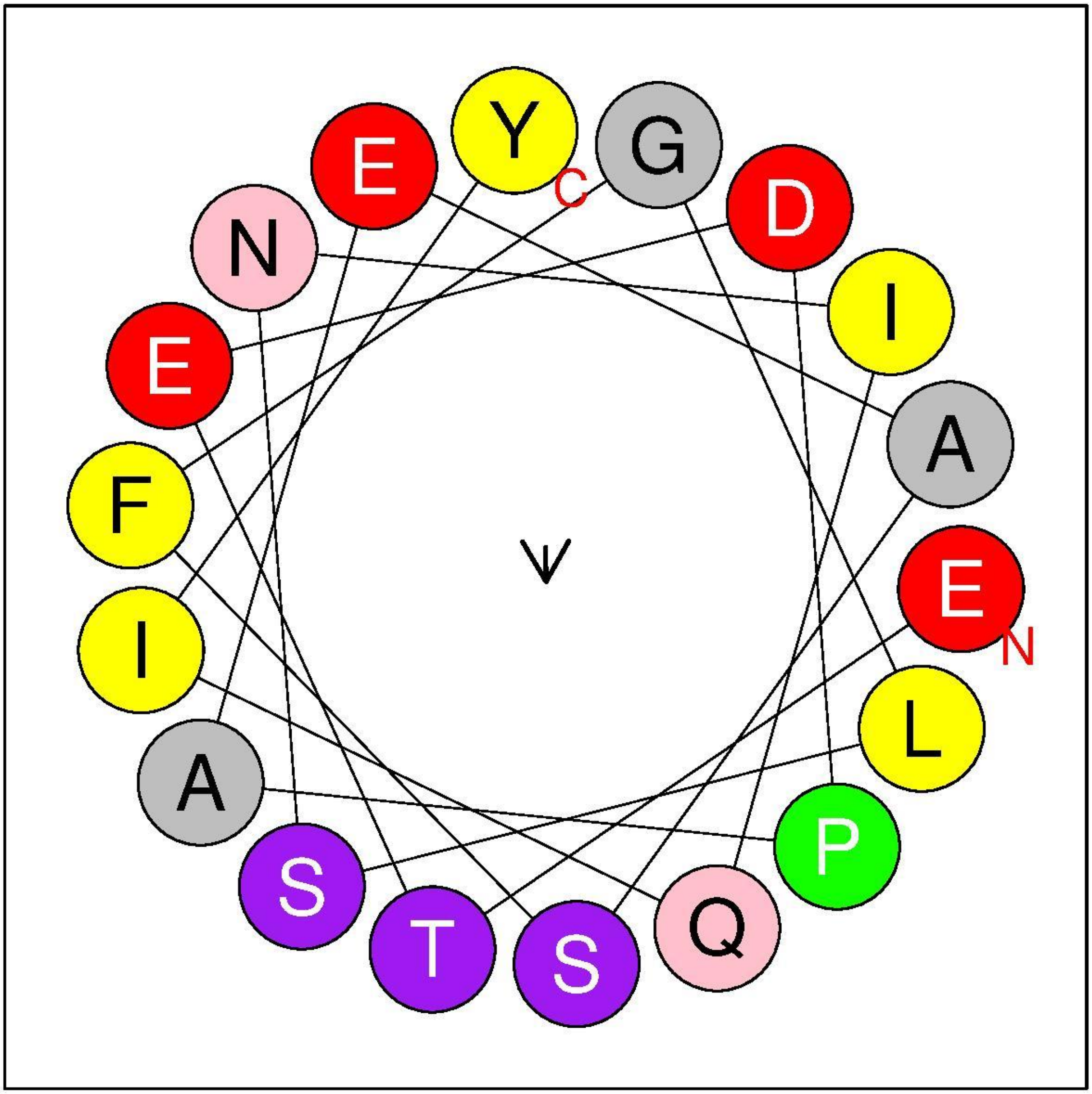

>TTHERM_00317390

LQDDPYENSYGISNLKIY

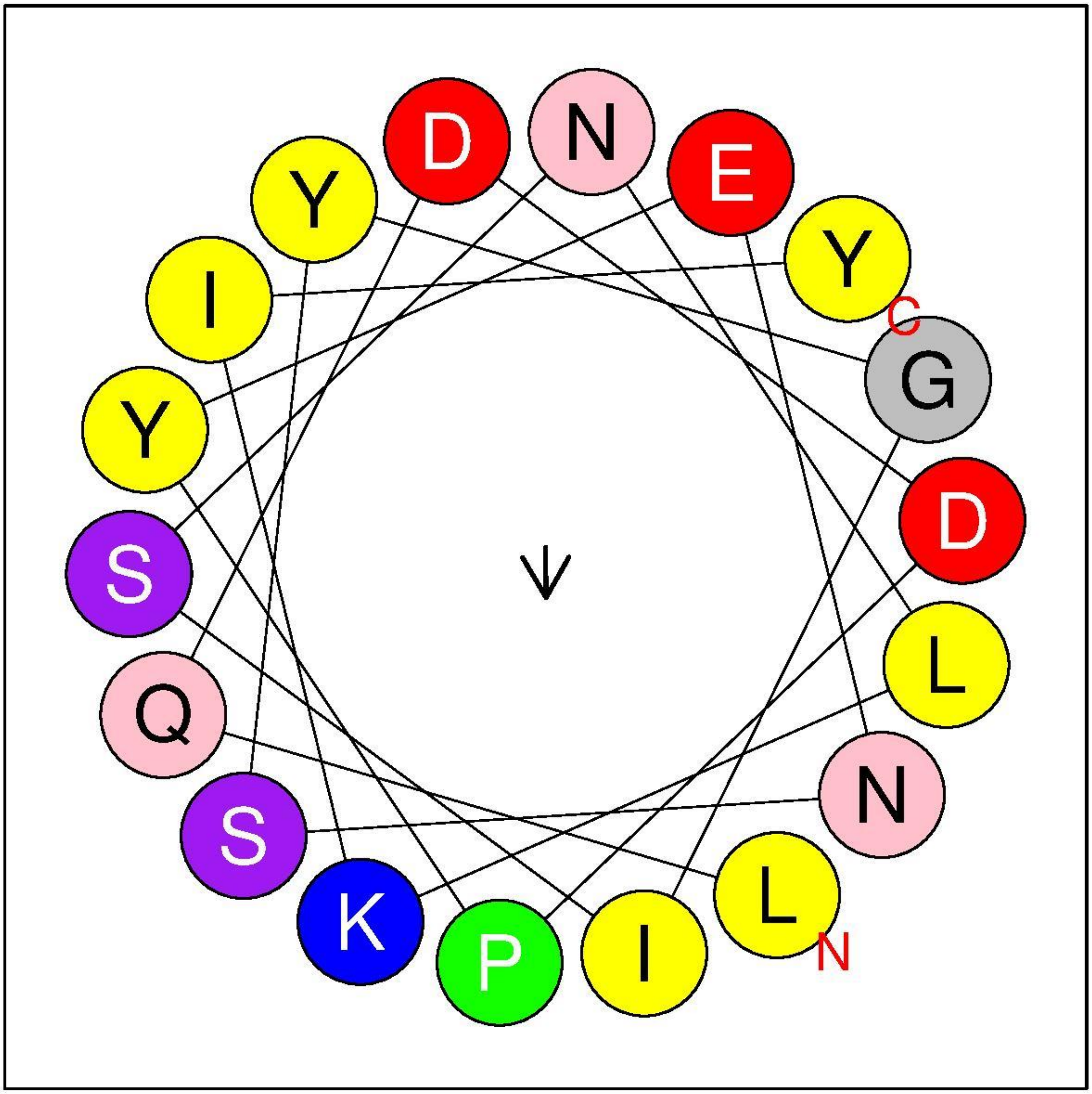

>TTHERM_00670750

KIQARVHFFDKWEGESLS

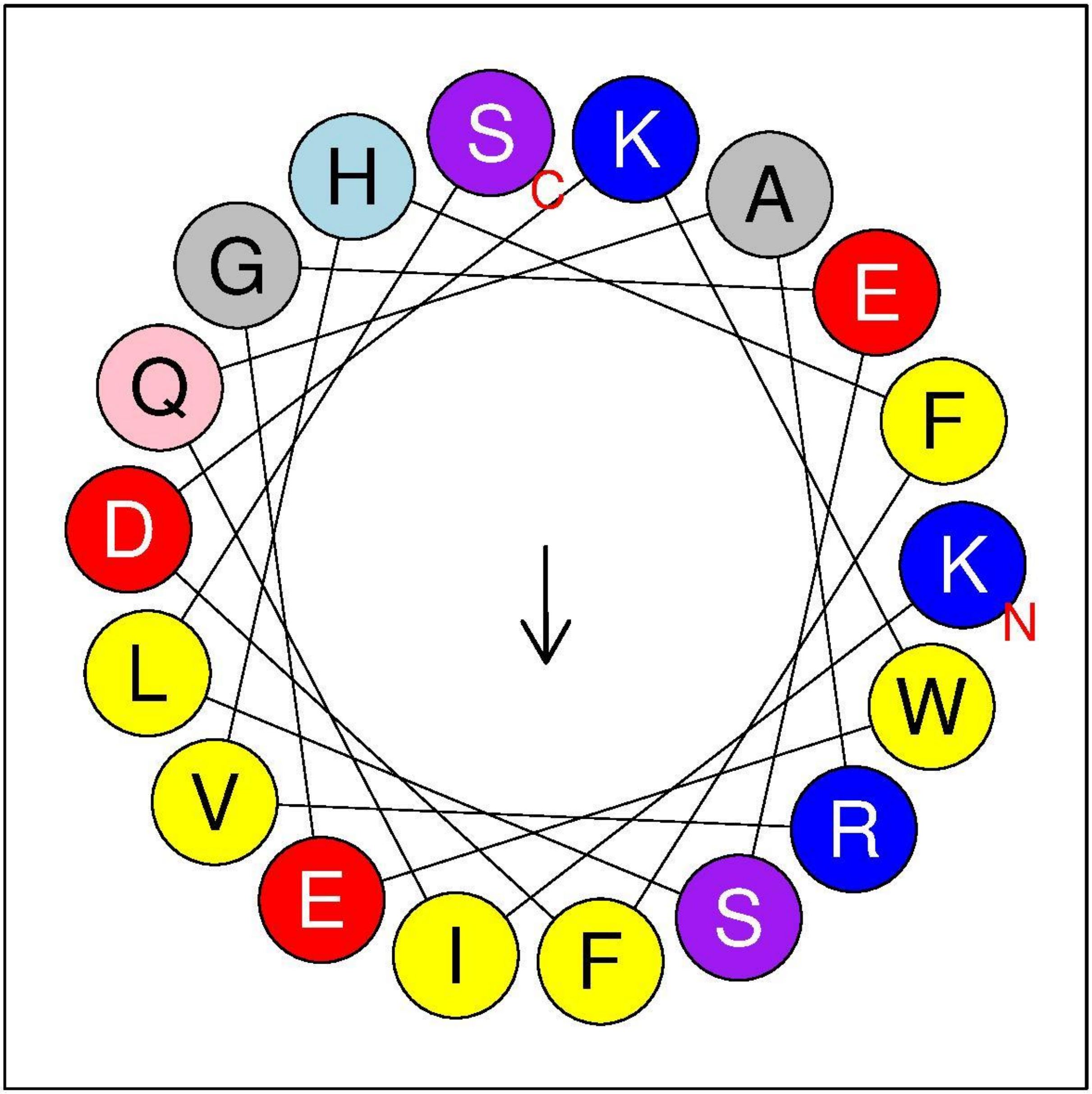

NLAKDRCLASFAIDDVEI

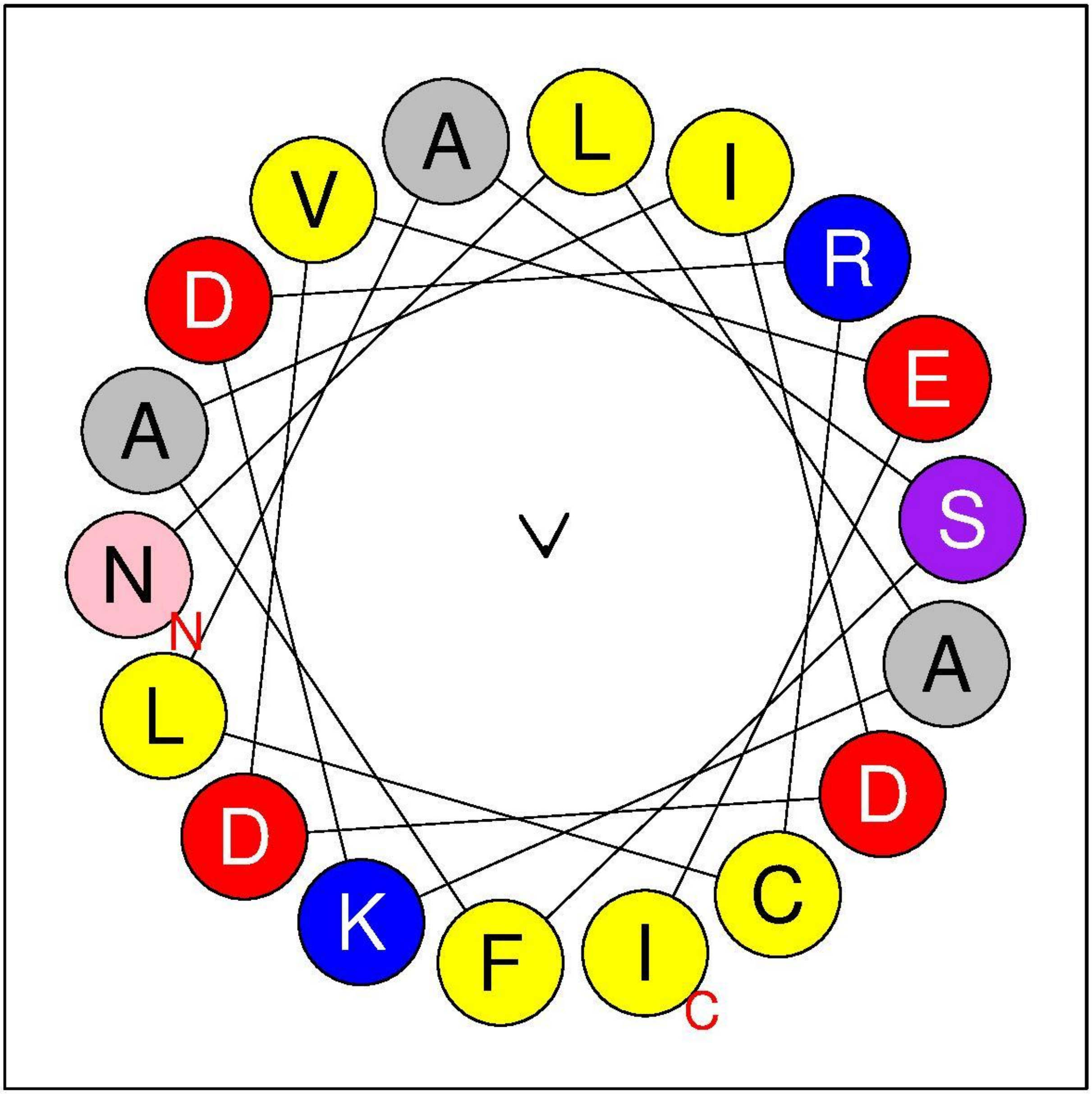

>TTHERM_01122800

NVKFNIHFFDDWTGELAF

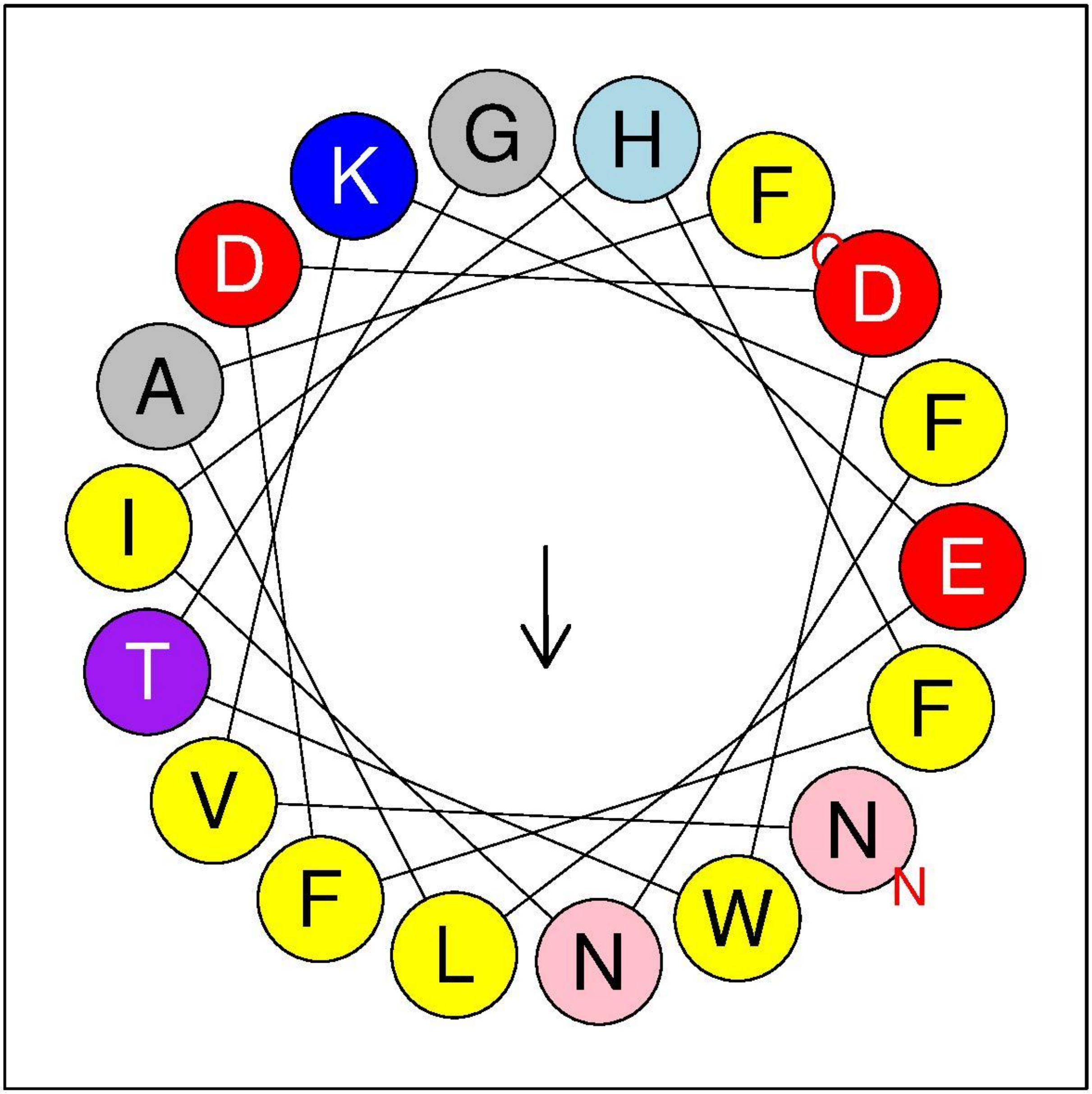

NLAKNSCDASWGIDDVQI

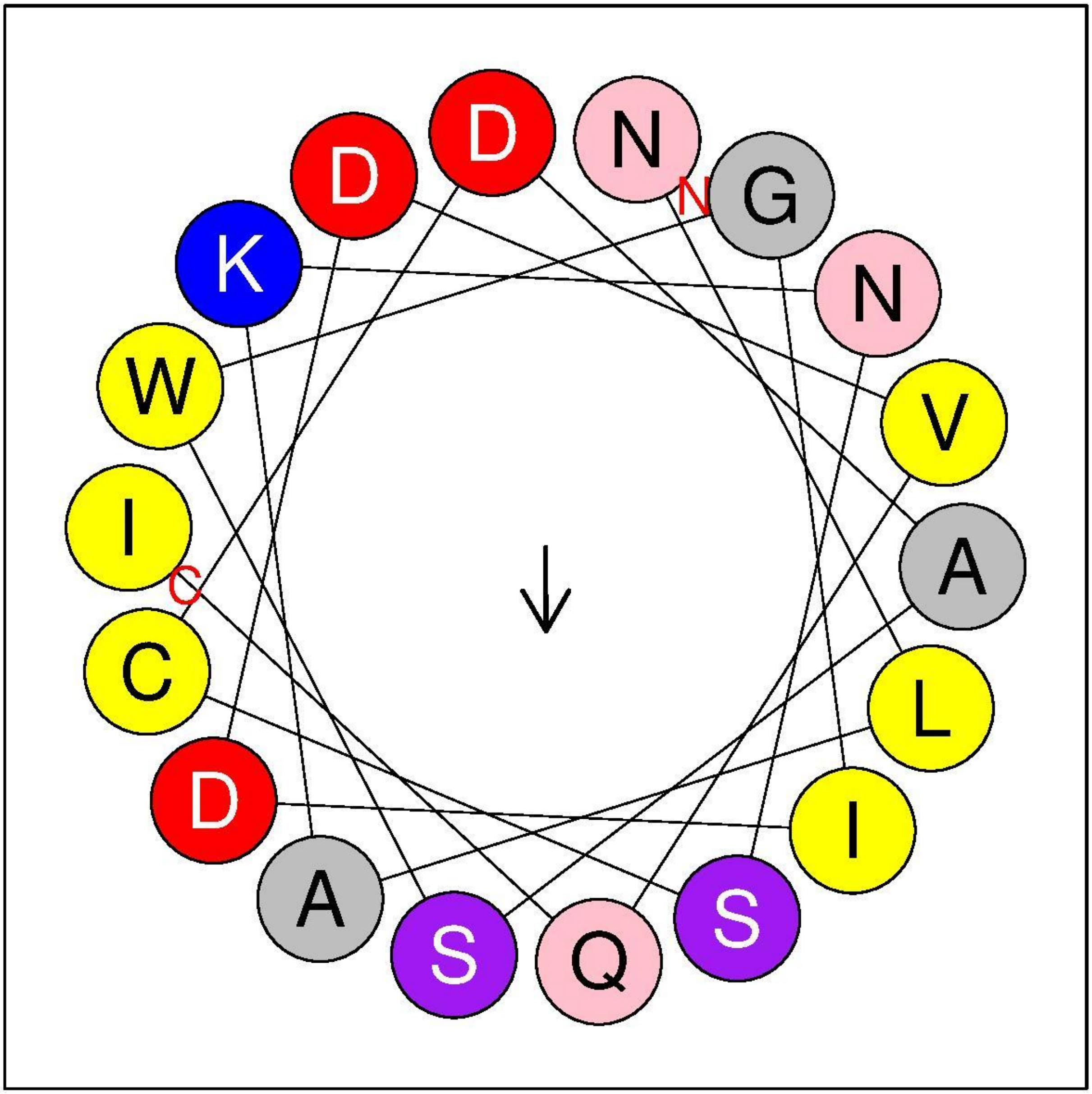

>TTHERM_01213910

KLSGTLHAFDNWEGEKLY

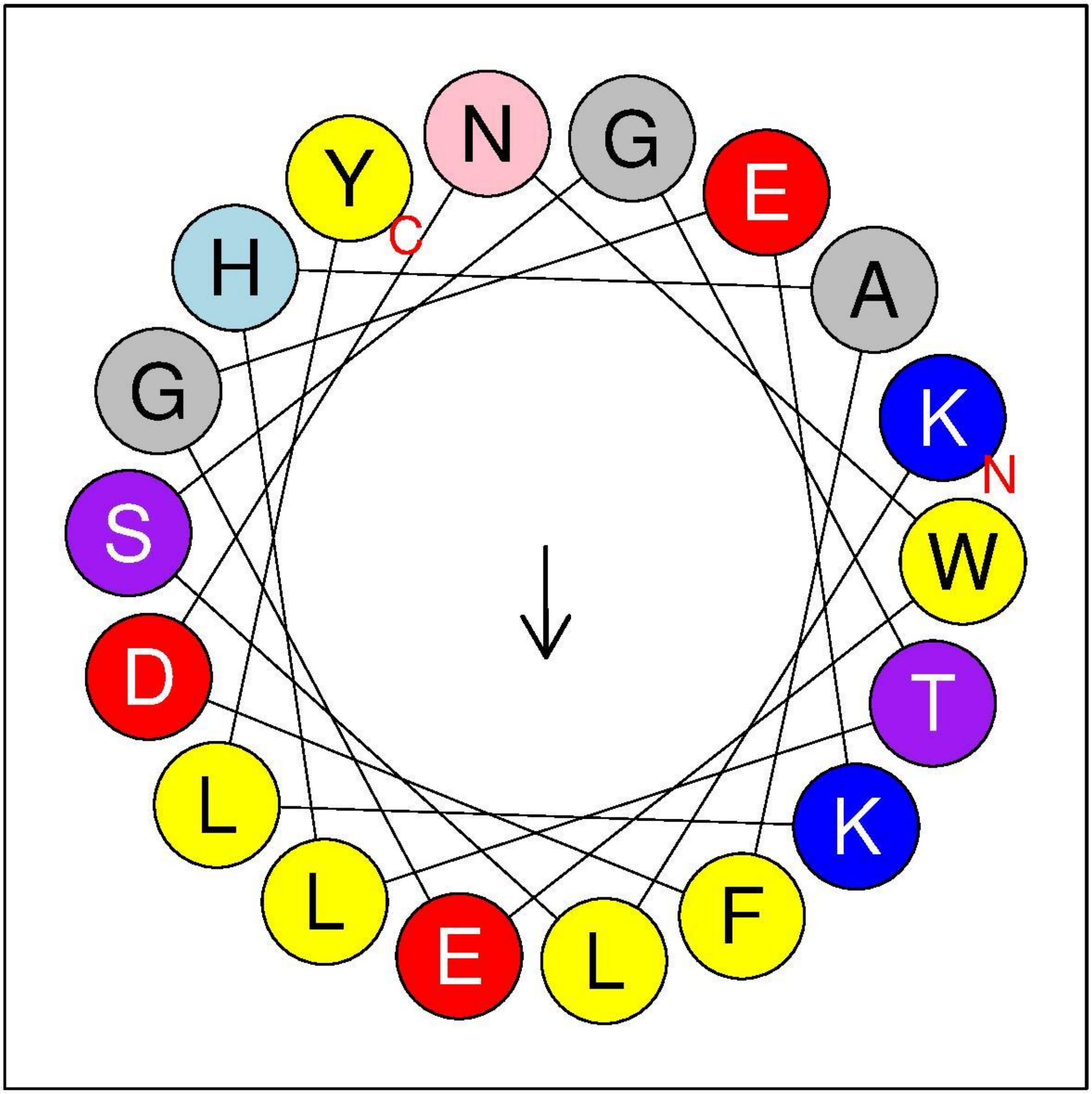

ELKKDSCDASYAIDDIIL

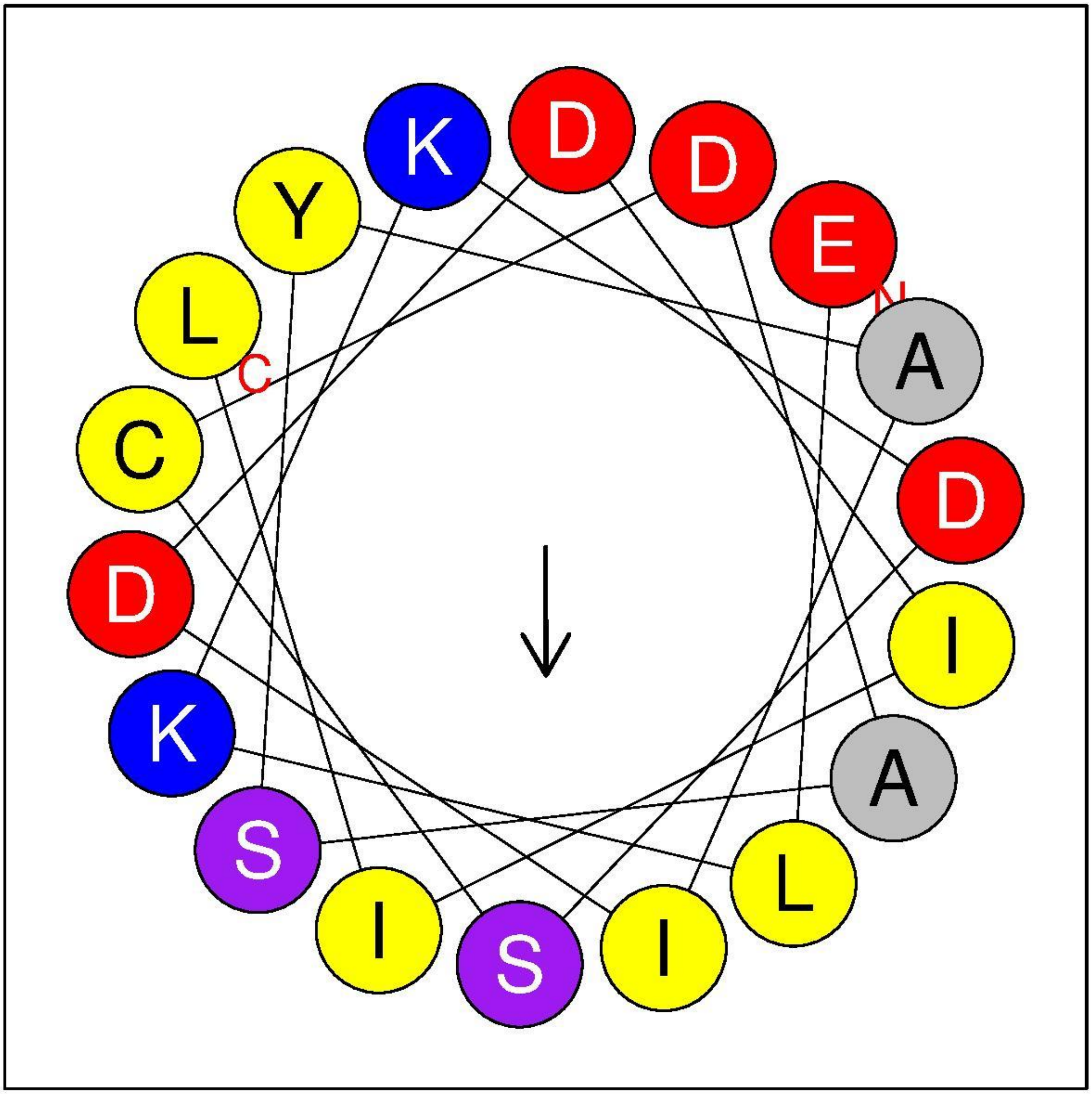

Supplement: S2 Fig — Predicted transmembrane/pore-lining residues of putative MDD subunits and their helical projections. For each protein, MEMSAT-SVM was used to predict membrane helices with the sequence of the full protein or with the signal peptide removed. Mdl1p and all putative MDD subunits are predicted to possess one or more 16-aa intervals that are transmembrane/pore-lining. For each protein sequence, a turn of α-helix (18 aa) including the interval predicted to be transmembrane/pore-lining by MEMSAT-SVM was used to generate a helical wheel (heliquest.ipmc.cnrs.fr). Residues are colored based on polarity and the arrow indicates hydrophobic moment (μH). (PDF) [file pgen.1010194.s002.pdf]
