# Supplementary material for: A novel membrane complex is required for docking and regulated exocytosis of lysosome-related organelles in Tetrahymena thermophila
Source: PLoS Genet. 2022 May 19;18(5):e1010194. doi: 10.1371/journal.pgen.1010194 (PMC9159632; doi:10.1371/journal.pgen.1010194)
Supplement: S1 Table — (DOCX) [file pgen.1010194.s007.docx]

S1 Table. *T. thermophila* strains used in this study

| Strain | Drug Resistance [Micronuclear Genotype (Macronuclear Phenotype)] | Exocytosis Phenotype | Mating Type |
| --- | --- | --- | --- |
| CU428 | *mpr1-1/mpr1-1*(mp-s) | exo+ | VII |
| B2086 |  | exo+ | II |
| MN175 | *mpr1-1/mpr1-1*(mp-r) | exo- | IV |
| SB281 | *chx1-1/chx1-1* (chx-s) | exo+ | III |
| UC300 |  | exo+ | II |
| IA267 | *chx1-1/chx1-1* (chx-s) | exo+ | III |
| F1 | *CHX/chx1-1;MPR/mpr1-1*(chx-r, mp-r) | exo+ | II |
| B*VII |  | exo+ | VII |
| CU428 UC300 GFP |  | exo+ | VII |
| CU428 UC300 FLAG |  | exo+ | VII |
| MN173 |  | exo- | IV/V |
| MN173 UC300 GFP |  | exo- | IV/V |
| MN173 UC300 FLAG |  | exo- | IV/V |
| CU428 UC300 CC→ AA  CU428 000193469-mNeon Igr1-mcherry  CU428 01213910-mNeon Igr1-mcherry  CU428 00141040-mNeon Igr1-mcherry  MN173 000193469-mNeon Igr1-mcherry  MN173 01213910-mNeon Igr1-mcherry  MN173 00141040-mNeon Igr1-mcherry |  | exo- | VII |
