# Supplementary material for: A novel membrane complex is required for docking and regulated exocytosis of lysosome-related organelles in Tetrahymena thermophila
Source: PLoS Genet. 2022 May 19;18(5):e1010194. doi: 10.1371/journal.pgen.1010194 (PMC9159632; doi:10.1371/journal.pgen.1010194)
Supplement: S2 Table — (DOCX) [file pgen.1010194.s008.docx]

S2 Table. Primers used for in this study.

| Vector | Primer | Sequence | Use |
| --- | --- | --- | --- |
| pNeo4-UC300-KO | UC300-1-Sac1FW | aaagagctctgtatattctaagttgatttactgct | To make 857 bp frag upstream of UC300 in pNeo4-UC300-KO/To make 897 bp frag uptream of UC300 in pNeo4mod-UC300-5’/3’ |
|  | UC300-1-Pst1Rev | aaactgcaggcttcacaaagtattagtcaaatct | To make 857 bp frag upstream of UC300 in pNeo4-UC300-KO |
|  | UC300-1-Xho1FW | aaactcgaggtaaataaactaggaatttgttgtat | To make downstream frag of UC300 in pNeo4-UC300-KO and pNeo4mod-UC300-5’/3’ |
|  | UC300-1-Kpn1rev | aaaggtaccatcaatgtcttttcaattatgtacat | To make downstream frag of UC300 in pNeo4-UC300-KO and pNeo4mod-UC300-5’/3’ |
| pNeo4mod-UC300-5’/3’ | UC300-1 Pme1Not1REV | aaagcggccgcgtttaaactctttgatcttttaatgctattttg | To make 897 bp frag uptream of UC300 in pNeo4mod-UC300-5’/3’ |
| pNeo4mod-eGFP-UC300 | UC300-1 sigseq EGFP FW | aaaGTTTaaacatgaaaaaataattttatttattaattttgtaattgatagtattttcatttgcaATGGTTTCTAAGGGTG | To make UC300 signal sequence + eGFP |
|  | EGFP kpn1apa1rev | AAAGGGCCCGGTACCACTAGTACTAGTCTTATATAATTCATCCATACCAAG | To make UC300 signal sequence + eGFP |
|  | UC300-1 Kpn1 FW | aaaggtacctatgatgtagaagatttaaggtaagat | To make UC300 without its signal sequence |
|  | UC300-1 Apa1 Rev | aaagggccctcaaaatcccttattaatacaacaaa | To make UC300 without its signal sequence |
|  | UC300-1-Nhe1rev endorep | aaagctagcaaatcccttattaatacaacaaattc | To amplify entire UC300 gene with eGFP tag with UC300-1 Pme1 FW as the forward primer |
| pNeo4mod-UC300-FLAG | UC300-1 FLAG FW | GATTACAAAGATGATGATGATAAAaatgtaaataaactaggaat | To make C-term UC300 + FLAG tag |
|  | UC300-1 FLAG rev | TTTATCATCATCATCTTTGTAATCtgggtctttcatttcatcta | To make N-term UC300 + FLAG tag |
|  | UC300-1 Pme1 FW | aaagtttaaacatgaaaaaataattttatttattaattttg | To make N-term UC300 + FLAG tag |
|  | UC300-1 Spe1 REV | aaaactagttcaaaatcccttattaatacaacaaa | To make C-term UC300 + FLAG tag |
| pNeo4mod-eGFP-UC300+CCtoAAmut. | UC300-1 Nhe1 CC to AA sub | aaagctagctcaaaatcccttattaatagcagcaattcctagtttatttac | To make CC to AA mutation in UC300 with UC300-1 Pme1 FW as the forward primer. |
| pPur4_Igr1-3mCherry2HA | NE_Igr1_gNheI_5f | GTGGCGGCCGCTCTAGAGgaaagatcattcttttgttag | To make C-term IGR1 + 3xmCherry2HA tag |
|  | NE_Igr1_gNheI_5r | CTGATGATCCTTGAGATCCGcaatttcttctgttgtttcttc |  |
|  | NE_Igr1_gXhoI_3f | cttatcgataccgtcgaCCgatagatcttttcaaataagtt |  |
|  | NE_Igr1_gXhoI_3r | TGGGTACCGGGCCCCCCCgcaaagtcatttgattgcg |  |
| pNeo5_000193469-2mNeon2HA | NE000193469_3f | AGATATCAAGCTTATCGATACCGTgtgttccaaaaatgatggc | To make C-term TTHERM_000193469+ 2mNeon2HA tag |
|  | NE000193469_3r | CCGGGCCCCCCCTCGAGGtatccacttcatagcttcc |  |
|  | NE000193469_5f | CTCCACCgcggTCCGCGAcaggaaatggatttcatcc |  |
|  | NE000193469_5r | TTCTTCACCCTTAGAAACCATTAAcatttaactgggctcttct |  |
| pNeo5_01213910-2mNeon2HA | NE01213910_3f | AGATATCAAGCTTATCGATACCGTacaaggatggatttggag | To make C-term TTHERM_01213910+ 2mNeon2HA tag |
|  | NE01213910_5f | CTCCACCgcggTCCGCGAaggatactctcatttaagg |  |
|  | NE01213910_5r | TTCTTCACCCTTAGAAACCATTAAacgaacacttaaaattatatc |  |
|  | NE12213910_3r | CCGGGCCCCCCCTCGAGGcttccaaaacaataaagcgc |  |
| pNeo5_00141040-2mNeon2HA.dna | NE00141040_3f | AGATATCAAGCTTATCGATACCGTctttcaaaacattgagttgc | To make C-term TTHERM_00141040+ 2mNeon2HA tag |
|  | NE00141040_5f | CTCCACCgcggTCCGCGAactgtaaaagctgctagtg |  |
|  | NE00141040_5r | TTCTTCACCCTTAGAAACCATTAAtttaacaaatatttctcttcc |  |
|  | NE10141040_3r | CCGGGCCCCCCCTCGAGGcatcgcaaccctctgag |  |
