# Supplementary material for: A novel membrane complex is required for docking and regulated exocytosis of lysosome-related organelles in Tetrahymena thermophila
Source: PLoS Genet. 2022 May 19;18(5):e1010194. doi: 10.1371/journal.pgen.1010194 (PMC9159632; doi:10.1371/journal.pgen.1010194)
Supplement: Attachment — Submitted filename: Letter with resubmission.docx [file pgen.1010194.s010.docx]

March 31, 2022

PLoS Genetics

To the editors,

We are delighted to return our revised manuscript entitled 'A novel membrane complex is required for docking and regulated exocytosis of lysosome-related organelles in Tetrahymena thermophila'. I hope we have satisfactorily responded to the reviewers’ and editor’s comments. We feel ourselves that our manuscript is considerably more informative as a result of this process. I would like to repeat my earlier expression of gratitude for the helpfulness of all the comments we have received, which has made this process much more rewarding than usual in my experience when submitting manuscripts for publication. Thank you!

Below please find detailed responses **in boldface** to all points raised:

Reviewer 1

1. Can the authors confirm that the partial MN175 phenotype that discriminates docking from fusion is obtained with the back-crossed line (maybe I missed something)? It would be elegant to check the phenotype with a GFP-transgene bearing the same Ser -> Phe amino acid change as Mn175, after gene replacement in the WT reference strain.

**We can confirm that, as noted by the editor, this analysis was done with the back-crossed line as stated in lines 188-192.**

**Because of the work and time required to make gene replacement lines, we were not able to introduce the mutant allele in the GFP-tagged strain, which we agree that this would be an elegant addition. Instead we focused on making the tagged replacement lines to address localization of a set of other proposed MDD subunits, as requested by reviewer #2 and described below.**

2. In Figure 2, a Muscle alignment of Mdl1p with homologs is shown. Yet I could not find in the legend or in the Methods, how these homologs were identified. I am guessing some kind of blastp search at NCBI? This should be specified clearly and accession numbers of the aligned proteins should be provided, at least in the figure legend.

**Thank you, all of this information has been added to the figure legend as requested.**

3. At several points in the manuscript, protein levels are compared on western blots, and it is stated that under one condition there is more protein, but there is no quantification. For example, in Figure 1E, with ‘a cross-reactive species’ -- which is vague indeed -- as loading control. Is there any way to make this (and other blots) more quantitative? So you can say there is 1.5X more protein or whatever. Similar situation in Figure 5B.

**We have added quantitation to these statements, in both the ‘Results’ text and in Figure legends. We have also clarified that the cross-reactive species seen in the Western blot is one that remains invariant between wildtype and mutant lines, which we had previously determined in studies of other mutants that affect mucocyst biogenesis. The reason we don’t simply use standard loading controls such as actin is because there are no commercially available antisera available that recognize the Tetrahymena proteins, due to their extensive sequence divergence from those in most eukaryotes.**

**In a related issue, the editors ask whether we can find a way to quantify docking in WT vs MN175 rather than relying on the qualitative analysis of the IF images. We have now done so by measuring IF signal that is restricted to the cell periphery, as a fraction of total cell signal, and we find that there is no significant difference between WT and MN175 (n=15). These data have been added to the relevant figure legend and the method described in Materials and Methods.**

4. Figure 2B, it would be helpful to reword the legend to make it clear that ‘WT’ and ‘MN175’ F2 pools refers to phenotype e.g. F2 pools with WT and MN175 phenotypes. I also stumbled at the beginning of the methods section “Genomic DNA preparation” it must be crystal clear that ‘WT’ refers to phenotype of F2 clones and not to WT cells. “32 wildtype and 32 separate F2 clones were pooled and grown in 25 ml of SPP overnight ” can be hard to understand for someone not working with these critters every day, please reword so it is perfectly clear that there is one pool of 32 F2 clones with WT phenotype and a second pool of 32 F2 clones with MN175 phenotype..

**This wording has been carefully re-worked, thank you, in both places.**

**The former now reads: “Comparison … between F2 pools with WT or MN175 phenotypes, of aligned sequencing reads around chr4: 5,364,873…”**

**The latter now reads: “Two pools of the F2 progeny, each containing 32 clonal isolates, were used to prepare genomic DNA. The first pool consisted of clones that were phenotypically wildtype, while the second consisted of clones that were phenotypically exocytosis-deficient, i.e., MN175. The clones in each pool were combined and grown in 25 ml of SPP overnight.”**

5. Line 211, there is a missing parenthesis

**Fixed, thank you**

6. line 232 missing article before 'putative' : “…as well as the presence of putative glycosylation site…”

**Fixed, thank you**

7. line 764 “33 ul” (Greek micron character should be used as elsewhere).

**Fixed, thank you**

8. Figure 1A an asterisk is mentioned but I could not see it in the image without blowing it way up on my computer screen. Furthermore, the legend states “the large majority of mucocysts (marked with *)” and thus it is a surprise to find only one that is marked on this image. Can the legend be reworded to convey the fact that only one mucocyst is so labeled in this image though a majority are similarly docked in the cell? Can the asterisk be made more conspicuous than black on black?

**We have changed the asterisk to white and increased the size, and we have marked additional mucocysts in the figure.**

9. DATA AVAILABILITY. The Illumina sequencing data (F2 pools) generated in this study should be deposited in GenBank and an accession number included in the manuscript.

**All data have now been deposited and an accession number is provided. We arbitrarily set the release date (when the data will be publicly visible) to 04-03-2023, but will make those data accessible as soon as the manuscript is accepted for publication. Until then, the editor/reviewers can see the submission under this link:**
<https://dataview.ncbi.nlm.nih.gov/object/PRJNA817605?reviewer=9fdg5gju5tnv6rn8in3e2shr4e>

Reviewer #2

Main comments

1. The gradient centrifugation and cryoEM analyses suggest that Mdl1p is in a protein complex, possibly with multiple copies of Mdl1p. In this situation it is an unlikely but still a non-zero possibility that the co-immunoprecipitating proteins are interacting with the FLAG tag rather than with Mdl1p itself. Such a potential artifact is typically addressed by using different affinity tags to immunoprecipitate the bait protein with one or more of the prey proteins.

**As suggested by the editor, we have responded to this by adding the transcriptional profiles where available for all of the other proposed MDD subunits, shown in S1B Fig. (For two of these subunits, those data are not available.) Also relevant to the point raised by the reviewer is our localization of three additional proposed MDD subunits, all of which were found to localize strongly to mucocysts (below).**

2. The authors tag one of the co-IPing proteins (01213910) with mNeonGreen and suggest it has a distribution that is consistent with it being associated with mucocysts, and their tips, but this remains largely speculative. The precise localization of 01213910 should be validated by IFA with known mucocysts markers.

**To address this we first tried to do precisely what the reviewer suggested, but we found that the pattern of localization in live cells was strongly attenuated when we fixed and permeabilized for IFA, even after trying a wide range of conditions. We therefore took an alternative approach and co-expressed the proposed MDD subunit, tagged with mNeon, together with an mCherry-tagged mucocyst content protein, Igr1p. To expand the approach, we did this for the original 01213910 but also for two other proposed MDD subunits, so that we would survey the different subfamilies of proteins within the proteomically-defined complex. We did this in both wildtype and MN173 cells, and all genes were expressed as endogenous replacements. Those data are now shown in the new Fig 7. Co-localization analysis was more complicated than for Mdl1p because: 1. the live cells could not be fully immobilized without producing stress-induced autophagy; 2. The cytoplasmic vesicles in MN173 cells are motile and the two colors are not captured synchronously on our microscope, and; 3. Expression of tagged Igr1p in MN173 induced the accumulation of a new compartment, which contains mucocyst markers but is larger and more irregular than the undocked mucocysts in MN173 expressing endogenous (un-tagged) Igr1p. Nonetheless, the analysis shows unambiguously that all three proposed MDD subunits are targeted to mucocysts, and that in the MN173 background they are no longer at the cell periphery but instead in cytoplasmic structures showing extensive co-localization with Igr1p. We also show, as noted above, that the available transcriptional profiles of all proposed MDD subunits matches that of *MDL1* as well as other known mucocyst-related genes (S1 Fig).**

**New text: 546-575**

**“Notably, all nine proteins, like Mdl1p, are predicted by MEMSAT-svm to possess one or more transmembrane/pore-lining domains, which are denoted and shown as helical projections in S2 Fig. We note that these are not classical hydrophobic transmembrane helices since all are predicted to include charged faces, which in some cases are extensive. To begin testing whether they constitute subunits of a complex, we asked whether three of the other putative subunits localize similarly to Mdl1p. In each case, we integrated mNeon at the C-terminus of the endogenous copy of the gene. In preliminary trials we found that tagged proteins formed punctate patterns strikingly similar to those of GFP-Mdl1p in live-cell imaging, but also that the puncta were lost upon fixation required for co-localization with mucocyst contents visualized by immunofluorescence. We therefore took an alternative approach, in both wildtype and MN173 backgrounds, of co-expressing the mNeon-tagged putative complex subunits with mCherry-tagged Igr1p, a mucocyst content protein. The co-localization analysis is less clear than for Mdl1p, particularly in the MN173 cells, because the cytoplasmic structures are in motion during live imaging and because the expression of tagged Igr1p appears to induce aberrantly large mucocyst-related structures. Nonetheless, all of the proteins were found to be unambiguously localized to docked mucocysts in wildtype cells, and present in cytoplasmic puncta overlapping with Igr1p in MN173 cells (Fig 7). These results are consistent with the idea that the proteins collectively form a complex on mucocysts, which we have named MDD for Mucocyst Docking and Discharge.”**

3. The complex containing Mdl1p was estimated to be 330 kDa, but limited substance was provided to support this estimate. It would be helpful to quantify the distribution of standards and Mdl1p to more transparently so the basis of the estimate.

**We have added more information on the estimation of complex size to the figure legend of S6 Fig. We re-evaluated this analysis and have revised downward our estimate of the MDD particle size, to ~275kDa. We share the reviewer’s frustration in the imprecision of the estimate, due to the difficulty in assigning peak gradient fractions both for Mdl1p (by Western) and especially the size standards themselves. During this revision period we also began to explore other and better approaches to estimating particle size, such as dynamic light scattering. Unfortunately, we do not currently have the resources to produce the amounts of protein needed for further structural analysis, though this is a high priority for us moving forward. The challenge is that isolating suitable amounts of the MDD complex from Tetrahymena for biochemical or structural analysis requires many weeks of work starting with 50-60 liters of culture. The isolated protein we have obtained has been used overwhelmingly to optimize conditions for EM (and cryo-EM analysis). Unfortunately, this means that we are unable to provide an informative stained gel of the complex. We also note that many of the MDD subunits are very similar in MW and will not be clearly resolved by SDS-PAGE.**

**As suggested by the editors, we have scanned the intensities of the Mdl1p signal across gradient fractions, and added a graph showing this distribution to S6A Fig.**

4. The authors suggest that the laminin domain of Mdl1p is oriented toward the lumen of the mucocyst, which indicates they are thinking that Mdl1p is embedded in the mucocyst membrane. However, it is unclear to this reviewer based on the data provided how a distinction can be made between their suggested scenario versus Mdl1p residing on the surface of the cell at sites of docking. Perhaps the authors are relying on the transcriptional profile, but the Mdl1p profile is not shown in relationship to known mucocyst or surface proteins.

**As noted by the editors, this point is addressed by data in the manuscript. A key point is that Mdl1p in MN173 cells (where the mucocysts are largely undocked) is no longer found at the cell surface but instead localized within the cytoplasm, where we show it co-localizes significantly with mucocyst markers. This is argued in lines 350-357. In the revised manuscript, we also follow the reviewer’s suggestion to show that the same is true for 3 other proposed MDD subunits.**

5. Point 3 also gets to another aspect of concern, namely the multiple instances where data is not shown. This reviewer is not familiar with the PLOS Genetics policies on data not shown but is a proponent of showing everything that is worth mentioning.

**Thank you, we have now added additional data as suggested.**

Minor
1. Given there is no direct evidence that Mdl1p forms a helical pore, it is best to describe the transmembrane domain as such alone.

**This is a delicate issue for us. We believe our data argue that Mdl1p is strongly membrane-associated, based on the detergent solubility experiments. Nonetheless it does not possess a classical transmembrane domain, while it instead has a domain predicted to be “pore-lining”. We have not done biochemical experiments on the other proposed MDD subunits, but each of them has such a predicted pore-lining domain but not canonical transmembrane domain. For this reason, we think that describing the domains as “transmembrane” could be misinterpreted.**

**What seemed most informative to us in light of these ambiguities, and following on the suggestion of the editors, we have now added helical wheel projections to show the predicted pore-lining domains of each of the MDD subunits. Those are found in S2 Fig. These show that all of the domains are predicted to have both hydrophobic and hydrophilic faces, which vary in their relative extent among the proposed MDD subunits. This is also spelled out in the new text:**

**(from line 546): “Notably, all nine proteins, like Mdl1p, are predicted by MEMSAT-svm to possess one or more transmembrane/pore-lining domains, which are denoted and shown as helical projections in S2 Fig. We note that these are not classical hydrophobic transmembrane helices since all are predicted to include charged faces, which in some cases are extensive.”**

**In consideration of this unsettled issue, we have also removed the sentence containing “transmembrane/pore-lining” from the manuscript abstract.**

Reviewer #3 

1. The image in Figure 2D is low in quality.

**As noted by the editor, the TIFF we have provided is of adequate quality.**

2. Lines 239-240 – “the mutation in MDL1 strain results in substitution of phenylalanine for serine at residue 147” – isn’t it a substitution of serine for phenylalanine?

**Thank you very much, we have clarified this. It now reads. “The mutation in *MDL1* in the MN175 strain results in substitution of serine by phenylalanine at residue 147”**

3. In the immunoprecipitation experiments, 140,000 x g was used to spin down the insoluble fraction (line 778). In Line 813, for the centrifuge speed value, please provide G Force value instead of rpm.

**Done**

4. Grt1p is “concentrated at one end of the rod, near the tip that fuses with the plasma membrane during exocytosis” (lines 108-109), while Grl3p is localized throughout the mucocyst and Mdl1p is at the tip. However, in Figure 3H, Grt1p and Mdl1p only partially co-localize. It needs to be clarified whether Grt1p is distributed over a wider area at the end of the mucocyst rod.

**Thank you. The reviewer is correct that Grt1p is distributed fairly broadly over the docking end of the mucocyst. We have added the following clarification to the Fig3 legend. “…cultures were fixed and immunolabeled with antibodies against the mucocyst tip protein Grt1p, which is distributed broadly at the docking end of the mucocyst.”**
